# Optical coherence tomography findings after surgery for sub-inner limiting membrane hemorrhage due to ruptured retinal arterial macroaneurysm

**DOI:** 10.1038/s41598-022-20949-3

**Published:** 2022-09-29

**Authors:** Ryosuke Hayamizu, Kiyohito Totsuka, Kunihiro Azuma, Koichiro Sugimoto, Taku Toyama, Fumiyuki Araki, Tomoyasu Shiraya, Takashi Ueta

**Affiliations:** Department of Ophthalmology, Graduate School of Medicine, Faculty of Medicine, The University of Tokyo, Tokyo, Japan

**Keywords:** Eye manifestations, Eye diseases

## Abstract

Ruptured retinal arterial macroaneurysm (RAM) can cause sub-inner limiting membrane (ILM) hemorrhage, leading to acute vision loss in the elderly. Vitrectomy has been established as an effective treatment to remove hemorrhage and facilitate visual recovery. Although optical coherence tomography (OCT) is useful for the diagnosis of sub-ILM hemorrhage before surgery, little is known about the postoperative OCT findings. Here, we retrospectively investigated the records of nine eyes of nine patients who underwent surgery for sub-ILM hemorrhage due to RAM rupture. On postoperative OCT, hyperreflectivity throughout the full thickness of the central fovea was observed in eight eyes (88.9%), and disruption of the ellipsoid/interdigitation zone (EZ/IZ) was observed in seven out of eight eyes (87.5%). The widths of the hyperreflectivity and EZ disruption gradually decreased. Visual recovery was least favorable in two eyes, in which the EZ line continuation did not recover until the final follow-up. The OCT findings corresponded to the hemorrhagic staining identified on fundus photographs in at least four eyes; as per the fundus photographs the findings persisted even after the hemorrhage was absorbed. In contrast, the OCT findings resembled the appearance before the development of a full-thickness macular hole, suggesting fragility caused by the RAM rupture.

## Introduction

A preretinal hemorrhage located between the inner limiting membrane (ILM) and the retinal nerve fiber layer is defined as a sub-ILM hemorrhage. Sub-ILM hemorrhage has different etiologies, commonly including by a ruptured retinal microaneurysm (RAM), Valsalva retinopathy, and Terson syndrome. sub-ILM hemorrhage can lead to sudden-onset, severe deterioration of visual acuity when the central macula is involved^1,2^. Although spontaneous resolution is possible for limited hemorrhage or in younger patients^3,4^, it can take several months. Prompt treatment is often desirable to avoid the irreversible toxic effects of hemoglobin and iron on the neural retina^2,4^. The key therapeutic strategy is surgical removal of the hemorrhage through pars plana vitrectomy with ILM peeling. This also helps to locate the hemorrhage definitively, that is, sub-ILM vs. sub-hyaloid. Another therapeutic option is drainage of the hemorrhage using Nd: YAG laser if the hemorrhage is detected within several days^4,5^. However, a higher risk of secondary epiretinal membrane formation has been reported^4,6^.

Optical coherence tomography (OCT) is a useful modality for diagnosing sub-ILM hemorrhage. In particular, the demonstration of both the posterior hyaloid membrane and ILM in front of the hemorrhage can exclude sub-hyaloid hemorrhage and facilitate an accurate diagnosis. In addition, high retinal reflectivity around the edge of the sub-ILM hemorrhage has been reported as a distinct OCT feature^7^. To date, postoperative OCT findings for sub-ILM hemorrhage remain unclear. In this retrospective review of patient data, we determined the characteristic postoperative OCT changes in sub-ILM hemorrhage caused by ruptured RAM.

## Methods

This retrospective study was approved by the institutional review board of The University of Tokyo Hospital (#2217), and conducted according to the Declaration of Helsinki.

### Subjects

We searched the records of patients who underwent vitrectomy for sub-ILM hemorrhage between April 2008 and August 2021 at the University of Tokyo Hospital. Eyes with pure sub-ILM hemorrhage over the central macula were included, and those with concomitant intra- and subretinal hemorrhage that affected the central vision were not included. Patients who did not attend the postoperative follow-ups at the University of Tokyo Hospital, and those with no available postoperative data, were also excluded. For the included patients, comprehensive ophthalmic examinations were conducted pre- and postoperatively, including best-corrected visual acuity (BCVA), intraocular pressure, slit-lamp microscopy, dilated-pupil fundoscopic examination, and spectral-domain OCT. BCVA was measured in decimals and converted to logMAR values for the analysis.

### Surgery

After informed consent was obtained from each patient, a 25G pars plana vitrectomy was performed by experienced ophthalmologists using CONSTELLATION (Alcon, Fort Worth, TX, USA). After core vitrectomy, the ILM over the hemorrhage was peeled to remove the sub-ILM hemorrhage, and the diagnosis of sub-ILM hemorrhage was confirmed. According to the surgeon’s preference, in some cases, the foveal ILM was preserved by peeling the ILM outside the fovea and sub-ILM hemorrhage was drained using a disposable 25G backflush instrument (Alcon) to minimize the risk of postoperative macular hole development^8^. Phakic eyes underwent combined phacoemulsification with monofocal intraocular lens implantation. After surgery, topical antibiotics and corticosteroids were gradually tapered.

### Spectral-domain (SD)-OCT

The majority of spectral-domain OCT (SD-OCT) images were obtained using SPECTRALIS (Heidelberg Engineering, Heidelberg, Germany), while a small portion of the images was taken using a 3D OCT-2000 (Topcon, Tokyo, Japan). Regarding SPECTRALIS, horizontal and vertical line scans passing through the central fovea were obtained using an automated eye-tracking software (TruTrack, Heidelberg Engineering). Line scans were created by averaging up to 100 B-scans within 30°, and each B-scan consisted of 768 A-scans. A built-in caliper tool was used to measure the width of the hyperreflectivity and EZ disruption. The width of hyperreflectivity at the central fovea was measured in the middle of the central foveal thickness.

### Statistical analyses

Statistical analyses were performed using the JMP Pro 14 software (SAS, Cary, NC, USA). The Wilcoxon rank-sum test was used to compare the numerical data between the two groups. Non-parametric analysis was appropriate due to the small sample size. Statistical significance was set at P < 0.05.

## Results

**Figure 1:**
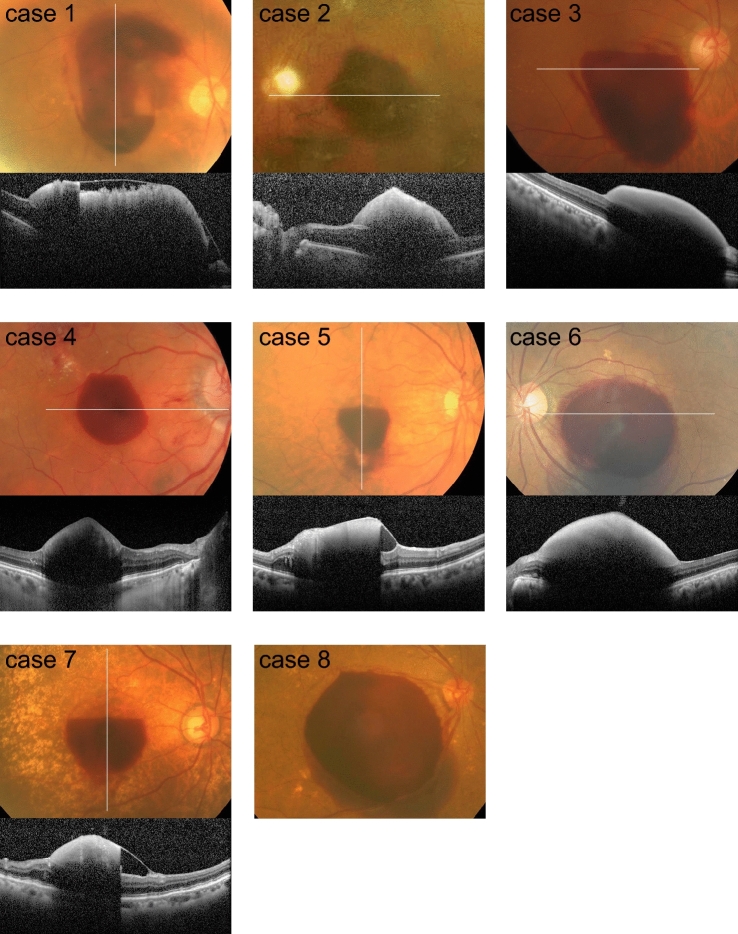
Fundus photographs and SD-OCT images of the included eyes before surgery. Preoperative images for case 9 were not available because of vitreous hemorrhage and dense cataract.

Nine eyes of nine patients were included in this study. Preoperative fundus photographs and SD-OCT images (Fig. 1) demonstrated that the sub-ILM space filled with hemorrhage covered the central fovea, explaining the cause of the acute visual disturbance. Preoperative images for case 9 were not obtained because of the presence of dense cataracts and vitreous hemorrhage. During surgery, the sub-ILM and not sub-hyaloid localization of the hemorrhage was confirmed. The underlying cause of sub-ILM hemorrhage was determined to be the ruptured RAM in all nine eyes.

Patient characteristics are shown in Table 1. The mean age was 84.7 (range 77–89) years. The numbers of male and female patients were 4 and 5, respectively. The mean interval from the reported onset of symptoms to the surgery was 11.4 (range 4–21) days. Mean BCVA before surgery was 1.20 logMAR (range 0.52–1.7). BCVA improved in all nine eyes 1 month after surgery. Further improvement was detected in eight eyes until the final follow-up. In seven of nine eyes, the final BCVA was 0.15 logMAR or better. Despite the older age of the patients, seven eyes were phakic before surgery, all of which were operated on and pseudophakic after surgery. There was no significant difference in the final BCVA between the eyes in which the ILM was preserved (mean; 0.19 logMAR) and those in which the ILM was conventionally peeled. (mean; 0.14 logMAR. P = 0.5, Wilcoxon rank-sum test).

**Table 1:** Characteristics of the included patients.

Case #	Age, years	Gender	Days from onset to surgery	BCVA before surgery, logMAR	Lens status before surgery	Foveal ILM	Follow-up after surgery, months	BCVA 1 month after surgery, logMAR	Final BCVA, logMAR
1	86	Male	4	1.52	Phakia	Preserved	12	0.82	0.15
2	83	Male	15	1.40	Phakia	Peeled	6	0.30	0.15
3	82	Female	13	0.52	Phakia	Peeled	6	0.40	0.15
4	77	Male	10	1.40	Pseudophakia	Peeled	6	0.40	0.10
5	86	Female	9	0.70	Phakia	Preserved	12	0.40	0.30
6	86	Male	9	1.15	Pseudophakia	Preserved	12	0.22	-0.08
7	87	Female	9	1.15	Phakia	Preserved	6	0.40	0.40
8	89	Female	21	1.22	Phakia	Peeled	3	0.22	0.15
9	86	Female	13	1.70	Phakia	Peeled	6	0.30	0.15

**Figure 2:**
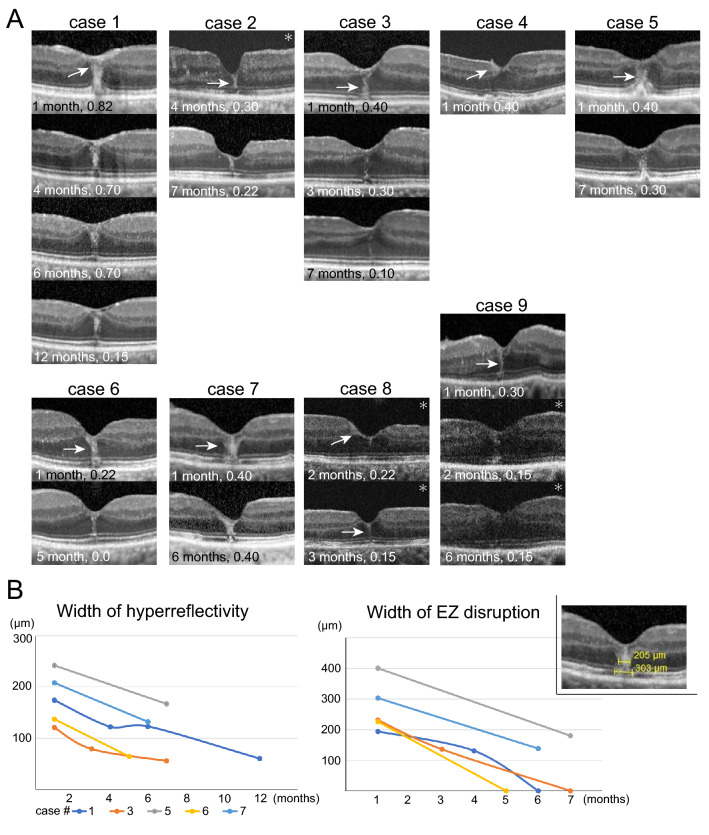
Postoperative SD-OCT images of the central macula. A majority of the images were taken using SPECTRALIS, and those marked with asterisk were obtained using 3D OCT-2000. (**A)** A vertical hyperreflective signal at the central fovea (arrows) was observed in all eyes, except in case 4. EZ/IZ disruption was observed in all eyes, except cases 4 and 8. In each image, the postoperative months of the image acquisition and a logMAR BCVA value are described. (**B)** Width of hyperreflectivity and EZ disruptions were measured in cases in which the SD-OCT images were taken using SPECTRALIS on two or more occasions postoperatively (cases 1, 3, 5, 6, and 7), showing the gradual fading of the OCT findings. Inset is the representative measurement of widths of hyperreflectivity and EZ disruption using a built-in caliper function.

The sub-ILM hemorrhage was successfully removed in all the cases. No significant perioperative complications required reoperation. In case 9, a macular hole developed one week after surgery, which was spontaneously closed a week later. Figure 2A shows the postoperative SD-OCT images of the central macula in each eye. Notably, in eight cases (88.9%), except in case 4, a linear hyperreflectivity was observed at the central fovea throughout the full thickness of the retina. In addition, in seven of the eight eyes (87.5%), except in case 8, hyperreflectivity was associated with the disruption of the ellipsoid/interdigitation zone (EZ/IZ). In Case 4, a mild hyperreflectivity was limited to the inner retina. In Case 8, mild hyperreflectivity at the foveola did not appear to accompany the EZ/IZ disruption. The hyperreflectivity and EZ/IZ disruption at the central fovea in seven cases gradually recovered but persisted for at least 3 months in at least six cases (85.7%), and over six months in five cases (71.4%). In particular, the width of the hyperreflectivity and EZ disruption was measured in five eyes (cases 1, 3, 5, 6, and 7) in which the SD-OCT images were obtained using the same machine (SPECTRALIS) on at least two occasions after surgery (Fig. 2B). The results indicated that in all five cases, the OCT findings faded over time postoperatively. In cases 1, 3, and 6, a recovery from the discontinued EZ line was observed 6, 7, and 5 months postoperatively. In cases 5 and 7, the EZ line disruption had continued until the end of the follow-up period, and the final BCVA was the least favorable among the eyes included in this study.

**Figure 3:**
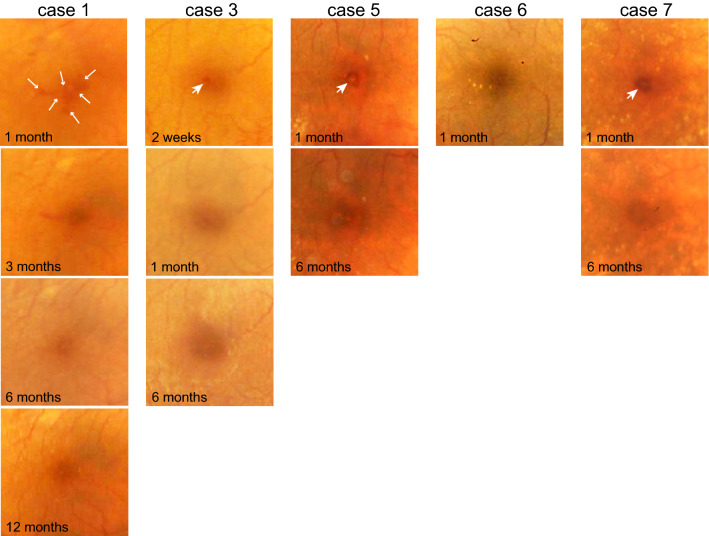
Serial postoperative fundus photographs show hemorrhagic staining at the fovea after surgery (arrows) that was absorbed during follow-up in cases 1, 3, 5, and 7. In case 6, hemorrhagic staining was not observed 1 month after surgery.

Postoperative fundus photographs available for five cases were evaluated to determine the possible sources of the characteristic OCT findings (Fig. 3). In cases 1, 3, 5, and 7, red-colored hemorrhagic staining was initially observed at the center of the fovea after surgery, which was gradually absorbed during follow-up. The area of hemorrhagic staining indicated on fundus photographs corresponded to the area of hyperreflectivity on SD-OCT. However, the OCT findings persisted even after the hemorrhagic staining had disappeared. In contrast, in Case 6, there was no hemorrhagic staining on fundus photographs, although the hyperreflectivity was evident on SD-OCT.

## Discussion

In this study, the characteristic postoperative OCT findings in eyes with sub-ILM hemorrhage caused by RAM rupture were described. The postoperative OCT findings for sub-ILM hemorrhage, regardless of the underlying etiology, have not been addressed previously. Hussain et al. reported 16 patients with sub-ILM hemorrhage due to Terson syndrome, RAM rupture, or Valsalva retinopathy, who underwent vitrectomy^2^. This study compared early and late surgical interventions for sub-ILM hemorrhage, although serial postoperative OCT images were presented for two eyes with RAM rupture. One eye showed mild inner retinal hyperreflectivity, whereas the other eye showed persistent hyperreflectivity and EZ/IZ disruption of the central fovea. Our study revealed that the latter is more commonly observed, and is a typical OCT finding after surgery for sub-ILM caused by ruptured RAM. In addition, our SD-OCT images during follow-up indicated that the OCT findings could take months to resolve, even after the absorption of the hemorrhagic staining at the fovea.

In eyes with hemorrhagic staining visible on postoperative fundus photographs, the area of hemorrhagic staining corresponded to the initial area of hyperreflectivity on OCT images. However, the same OCT findings were observed in the eyes even after the hemorrhage was absorbed and in those in which no apparent hemorrhagic staining was present. Moreover, the same OCT findings were observed in the eye in which the postoperative macular hole developed and then spontaneously closed. Hence, hemorrhagic staining alone may not be sufficient to explain the synthesis of the OCT findings. In contrast, a previous study reported OCT findings before the development of full-thickness macular holes, and similar OCT findings comprising a vertical hyperreflective line with EZ/IZ disruption at the central fovea were frequently observed^9^. Therefore, we speculate that the rapidly produced high pressure in the sub-ILM space at the time of RAM rupture may render the central fovea more fragile and change its reflective properties. In line with this, a higher risk of macular hole development after RAM rupture has been reported^8,10,11^. Additionally, a study has also reported that sub-ILM and subretinal hemorrhage are risk factors for the development of a macular hole in patients with ruptured RAM^11^.

This study has several limitations and our results should be interpreted with caution. First, because of the retrospective nature of this study, SD-OCT images and fundus photographs were not available regularly. In addition, due to the same reason, the OCT images were not taken with a specific focus on the hyperreflectivity, including 3D structural evaluations, although the hyperreflectivity may not be a perfect cylinder shape. These issues led to a difficulty in assessing exactly when the OCT findings disappeared, or whether or not the visual recovery coincided with the recovery from EZ/IZ disruption. In addition, they could also prevent the accurate measurement of the width of the hyperreflectivity and EZ disruption. However, a consistent decline in the width of hyperreflectivity and EZ disruption was observed in all evaluated cases, suggesting the magnitude of the potential inaccuracy may not be significant. Second, the central visual field, another important examination of the central vision, was not evaluated. Third, a relatively small number of eyes were included because sub-ILM hemorrhage without intra- or subretinal hemorrhage in the central macula is uncommon. However, a hyperreflective stress line, frequently with EZ/IZ disruption, was observed at a high rate after surgery. Lastly, we did not find cases with underlying etiologies other than RAM ruptures. In contrast to Terson syndrome or Valsalva retinopathy in which the retina is otherwise healthy, the retina with RAM may be more vulnerable due to long-standing insults caused by uncontrolled hypertension and older age, which may have contributed to the OCT findings in this study. Indeed, previous reports have suggested a better visual prognosis after sub-ILM hemorrhage in patients with Valsalva retinopathy^2,12^.

In conclusion, the characteristic OCT findings after surgery for sub-ILM hemorrhage caused by RAM rupture were confirmed in this study. Vertical hyperreflectivity with EZ/IZ disruption on SD-OCT may represent central fovea fragility and may be mechanistically related to a hyperreflective stress line commonly observed before the development of a macular hole.

## Data Availability

Data generated during the current study are available from the corresponding author on a reasonable request.
